# “My Husband Is a ‘Mama’s Boy’”: Women’s Views on Male Engagement in Maternal, Newborn, and Child Health in Western Kenya

**DOI:** 10.3390/ijerph22010125

**Published:** 2025-01-19

**Authors:** Robsan Tura, Nema C. M. Aluku, Sato Ashida, William T. Story

**Affiliations:** 1 Minnesota Department of Health, St. Paul, MN 55164, USA; 2 Africa Community Leadership and Development, Nairobi, P.O. Box 24619, Kenya; naluku@aclad-hq.org; 3 Department of Community and Behavioral Health, College of Public Health, University of Iowa, Iowa City, IA 52246, USA; sato-ashida@uiowa.edu (S.A.); william-story@uiowa.edu (W.T.S.)

**Keywords:** Kenya, maternal, newborn, child health, male engagement, qualitative methods

## Abstract

There is an increasing global acknowledgment of the critical role that men have as key partners in maternal, newborn, and child health (MNCH). Most male-engagement initiatives do not address the perceived benefits and risks that women may experience because of increased male participation in MNCH, especially in Kenya. The aim of this study, therefore, is to qualitatively assess how women perceive and experience increased male engagement in MNCH in western Kenya. Using a phenomenological approach, 53 women (35 mothers and 18 mothers-in-law) were purposively selected from seven communities in Kakamega County and participated in seven focus group discussions (FGD) in November and December 2020. Our findings suggest that the level of support for increased male involvement in MNCH varies depending on the type of participant. While mothers-in-law were less supportive of increased male involvement, mothers were more supportive of male involvement that fosters gender equity, such as joint decision making. Most mothers-in-law argued that women are ‘naturally’ closer to children, that men cannot care for children and their involvement may harm children, and men’s involvement may give men more control over women’s spaces. These findings show that programmatic initiatives to enhance male engagement in MNCH must consider mothers-in-law’s concerns and leverage mothers’ desire to engage men in a gender-equitable way.

## 1. Introduction

Although evidence related to the benefit of engaging men in maternal, newborn, and child health (MNCH) has ignited increased programmatic and research interests, it is not entirely clear how these new developments are viewed by women. Women’s perspectives have largely been ignored in the male engagement research agenda [1]. The recognition of women’s views on increased male involvement in MNCH, which is a product of their sociocultural environments, is missing from the male engagement literature [2,3,4,5,6]. The anticipated benefit from engaging men in MNCH depends on, among other factors, whether and to what extent women want men to engage.

If men engage in MNCH to reaffirm their masculine identity by exerting control over women and disrupting secure social spaces for women, then women may not benefit from their involvement [7]. For example, if men accompany their female partners to health facilities to control their movement and actions, then women will not have the chance to meet other women and engage in conversations about their pregnancy, childbirth, and childcare experiences [2]. In addition, male presence at the health facility may restrict women from discussing important issues with their healthcare providers, particularly issues related to family planning and contraceptive use for fear of intimate partner violence [2]. Furthermore, if male engagement strategies require women to bring their male partner to antenatal care (ANC) visits or require healthcare providers to give preference to couples who attend ANC together, as it is in many low- and middle-income countries (LMICs) [8,9], then male engagement initiatives may unintentionally prioritize ANC for women who are married or increase the burden on women to involve their partner. It is possible for such well-intended initiatives to pose a challenge to women’s autonomy in cases when women do not want their male partner to attend ANC due to diverse reasons, such as intimate partner violence or controlling behavior [9,10]. In societies where gender norms have historically excluded men from MNCH responsibilities, it can have negative impacts if more pressure is placed on women to attend ANC appointments with their partners [11,12]. Such male engagement strategies can also result in the stigmatization of women who are not married or do not have a steady partner [3].

In western Kenya, the site of the current study, gender-based role differentiation and male domination are prevalent [9,10]. Moreover, in most communities in the region, including the seven communities where this study was conducted, the kinship system is patrilineal, and the organization of the kinship structure around patrilines often marginalizes women [13]. For example, men are often in charge of decision making and are seen as the breadwinners, and women have less decision-making power [4,14], which prevents men from engaging in MNCH in a meaningful and gender-equitable way.

This study, grounded in Connell’s theory of masculinity, explores the influence of hegemonic masculinity on MNCH behaviors [15]. Hegemonic masculinity emphasizes traits such as dominance and control, often excluding men from caregiving roles and reinforcing the patriarchal norms described above [15]. This is particularly relevant to the understanding of how traditional masculinity limits men’s involvement in MNCH and how these norms are upheld by women, especially mothers-in-law, or how traditional masculinity is challenged by younger generations. Therefore, this study seeks to understand how the perception of hegemonic masculinity among women (both mothers and mothers-in-law) in western Kenya affects male engagement in MNCH. Women’s views of masculinity may, in turn, affect their willingness to embrace greater male engagement in MNCH, as well as their ability to benefit from initiatives to increase male engagement.

## 2. Materials and Methods

### 2.1. Study Design, Participant Selection, and Recruitment

The study was conducted in Kakamega County of western Kenya. The county has a population of 2 million and is divided into 12 sub-counties [16]. This study employed a phenomenological approach using focus group discussions (FGDs) to understand women’s lived experiences and perceptions of increased male engagement in MNCH in 7 of the 12 sub-counties of Kakamega County. Women were identified through the village master list obtained from local leaders, and FGD facilitators screened all identified women for eligibility to participate. Women were selected purposively using the following inclusion criteria: age 18 or older, having a child or having been pregnant in the last 12 months (mothers only), being a mother of a man or woman who had a child in the last 12 months (mothers-in-law), availability and willingness to participate in the study, and ability/capacity to consent to participate. The exact age of the participants was not recorded; therefore, it is possible that some of the participants were married or had become pregnant before the age of 18. A decision was made to include all women regardless of their age of marriage or pregnancy because these practices are common in this part of western Kenya, and male involvement can mitigate or exacerbate the challenges that adolescent mothers face [17]. The study team also decided to exclude those who were pregnant at the time of the FGDs due to their increased vulnerability to COVID-19 and pandemic-related restrictions in place in the study area during data collection (November to December 2020). Enumerators contacted the participants door-to-door to request their consent, and all those invited agreed to participate. A total of 53 women (35 mothers and 18 mothers-in-law) participated in the FGDs. In total, seven FGDs were completed—two with mothers-in-law and five with mothers who were either lactating or had a child in the last 12 months at the time of the study. All participants were married to and lived with a male partner at the time of the interview. In total, 34 were from rural villages, and 19 from urban or small-town settings. One mothers-in-law FGD was conducted in each setting (rural/urban), along with three mothers’ FGDs in rural areas and two in urban areas.

### 2.2. Data Collection

An open-ended thematic FGD guide was designed and used in all FGDs. The FGD guide was designed to explore women’s own experiences and views on increased male engagement in MNCH, their views on traditional gender roles, and the extent to which they [women] want men to engage in MNCH. The interview guide was flexible and allowed for pertinent issues that arose during the FGD to be further probed. The interview guide was pilot tested in one of the study communities using the local language (Luhya), which helped to reframe and clarify the questions and use more culturally and linguistically appropriate words. The final English version of the FGD guide is included in the Supplemental Materials Annex S1.

Four FGD facilitators and four notetakers received in-depth qualitative data collection training, which provided facilitators and notetakers essential skills, such as effective probing techniques, managing group dynamics, and addressing sensitive topics. The training also emphasized ethical considerations, ensuring respectful and culturally appropriate engagement with participants during the FGDs. All FGDs were conducted in the local dialect—Luhya. All FGD facilitators were from the study area and fluent in Luhya and English. Data were collected over a period of eight days between November and December 2020. Each FGD consisted of 6–11 participants and the discussion in the groups lasted 1.5 h on average. FGD facilitators completed each FGD when a saturation point was reached (i.e., the point at which no new information is being revealed). The FGDs were held in community venues convenient to the participants and audio recorded alongside hand-written field notes. No one was present during the FGDs besides the FGD participants. As part of data quality assurance measures, two meetings with facilitators during data collection were held to review data collection processes and to resolve any inconsistencies or challenges encountered. Audio recordings were first transcribed into the local language (Luhya) and then translated into English before analysis. The decision to code the transcripts in English was made because it is the shared language of the authors. Transcription and translation from Luhya to English were carried out by local professional translators. When the English translation was in doubt, the research team back translated the text to ensure accuracy and preserve the meaning of the words during the translation process.

### 2.3. Data Analysis

Data were analyzed using a combination of content and thematic analysis to examine views and opinions, and explore unknown phenomena [18,19]. These two techniques have been used together in previous studies to help bridge between the empirical data and theoretical literature while paying attention to the content, interaction, and context of the data [18,20,21]. During content analysis, specific words and phrases were identified within the transcripts. This step primarily focused on exact word or phrase matches rather than contextual meaning. For example, when examining how women endorse traditional masculinity, content analysis identified how often they associated a “real man” with sexual power, fertility, or the ability to provide for his family. It also captured how frequently women reported their male partners’ involvement in MNCH activities. Thematic analysis focused on the latent content expressed through words and phrases, uncovering deeper meanings and significance in the data. For instance, the way in which women’s views on traditional masculinity varied between mothers-in-law and mothers was explored, as well as the reasons behind men’s lack of engagement in MNCH.

Using these two strategies, an inductive coding approach was employed to generate a codebook. In vivo codes were also generated based on participants’ own words, highlighting data that represented the participants’ ideas and context. Once the codebook was finalized, the transcripts were analyzed by the lead researcher using NVivo 11. The final code structure was used to report the findings, and, where relevant, verbatim quotations from the FGDs were included to illustrate key points or themes.

## 3. Results

### 3.1. Women’s Experience with Male Engagement in MNCH

During FGDs, participants were asked about their male partners’ engagement in MNCH. Specifically, whether their husbands or partners have ever accompanied them to health facilities (for prenatal, antenatal, delivery, and postnatal care), assisted in household chores, and took care of children (e.g., changing diapers, bathing, feeding the child, putting the child to bed). Although several women, especially mothers, reported that men’s engagement could facilitate easy access to antenatal and postpartum care, they indicated that their husbands or partners were never involved in MNCH care and barely provided them with financial or transportation support for antenatal, delivery, and postnatal care. When probed for the reason why men are reluctant to engage in MNCH, especially attending the health facility with their partners, participants reported several reasons including social pressure (e.g., fear of being ridiculed by peers, fear of being seen as feminine), influence from mothers-in-laws, being in a polygamous relationship, religious beliefs, and living in a rural area.

Social norms and peer pressure:

*“In our community, many men, including my husband, do not help their wives because they fear being laughed at by their friends. They will be seen as fools by people in society.”*

(A mother from FGD 5)

*“My husband did not do those tasks because he claims that those are feminine duties. When I ask for his help, he would say he would rather hire a maid for me…and you as a wife should be very careful about this one, your husband can even decide to marry her. Other people would also claim that I’ve used charms on him.”*

(A mother from FGD 3)

Influence from mothers-in-law:

*“My husband never even helps in washing the child. My husband is a ‘Mama’s boy.’ He stays at his mother’s place almost all the time and whatever he is told by his mother is what he does. My mother-in-law has not been forthcoming in educating her son to understand his role as a husband…. She does not want him to help me with any household chores and to accompany me to the hospital.”*

(A mother from FGD 4)

*“I hear my mother-in-law and my own mother saying pregnancy and house chores are for women. Personally, I do not agree that these tasks should be for women only.”*

(A mother from FGD 3)

Being in a polygamous relationship:

*“My husband is a polygamist (is in a polygamous relationship), I happen to be his sixth wife, so if he didn’t take [care of] the rest of his wives, would he have taken [care of] me? And I think my co-wives would be mad at him if he [prioritized] me, that’s what he feared.”*

(A mother-in-law from FGD 1)

Religious beliefs:

*“My husband’s religion played a part in him not to help me as he attends a church that believes in God’s healing alone. So going to the hospital was not on the agenda.”*

(A mother from FGD 2)

Living in the rural area:

*“While I was still living in an urban center, people in the community found it normal. But when we relocated to the village, it became a problem because people started underestimating my husband causing him to stop accompanying me for health care visits.”*

(A mother from FGD 5)

Participants who reported experience with male engagement defined engagement as accompaniment to healthcare, financial support, arranging transportation to the health facility, helping with household chores, and taking care of children (e.g., changing diapers, feeding children, doing laundry). They also reported the reasons that motivated their partner to be engaged, such as love, care, health of the child and wife, and fear of potential pregnancy complications.

*“When a husband gets involved with the children, you get encouraged and also know that love exists in the marriage in that when you are held up, you know that the husband will take care of the children.”*

(A mother from FGD 1)

*“Men of our days never used to take women to the hospital, not like [men] nowadays. Couples nowadays love each other a lot.”*

(A mother-in-law from FGD 2)

*“I think it is because he was part of the pregnancy, and it was our child and he therefore has to take care of me during that period.”*

(A mother from FGD 3)

### 3.2. Women’s Perspectives on Male Engagement

In addition to their experience with men’s engagement in MNCH, participants were also asked whether they would want their husbands or partners to be directly engaged in issues of MNCH. Themes emerging from the analysis show women’s views of increased male engagement in MNCH reflected stark differences between mothers-in-law and mothers at the time of the study. A summary of the desirability of male engagement in MNCH by women is presented in Table 1.

#### 3.2.1. Mothers-in-Law

In general, the FGDs conducted with mothers-in-law suggested that they strongly favor gender role differentiation, including roles related to MNCH. Most mothers-in-law indicated that their husbands or partners never engaged in MNCH beyond the provision of financial support and neither did they (women) want their husbands or partners to be more involved beyond providing money and transportation support. Other mothers-in-law questioned why men were being asked to be involved in MNCH beyond providing needed financial resources.

*“I have seen my neighbor’s husband doing domestic work…. The husband cleans the baby and does the baby’s laundry. But for me, my husband can never do a baby’s laundry. What he does for his children is to pay school fees, which is good. As long as he provides the fare, I am good to go, and it does not hurt me. For me, I do not understand why men have to be involved in women’s roles. Is it because women can no longer take care of their house, children, and their pregnancies?”*

(A mother-in-law from FGD 1)

When this respondent was probed about what she thought about her neighbor doing domestic work, the respondent said, “He is just doing that because he is too lazy even to do farm work and provide for his family like a man. His wife is the one who provides for the family.”.

Some mothers-in-law also argued that men should not be involved in MNCH because they do not have the natural ability to get pregnant and give birth. According to the mothers-in-law, it would be unfair to expect men to be actively engaged in MNCH and it is natural for women to take care of their pregnancy.

*“It is natural that we women get pregnant and give birth; men do not. So, I think it is not natural if we expect our husbands to play the role that women have been created to play. Also, when they get involved, they start controlling their wives’ movements and how much the women pay at the clinic”*

(A mother-in-law from FGD 2)

One participant agreed:

*“I also think that what my sister just said is right. It is how God created us. It is women who get pregnant and give birth, so naturally, it is our obligation to care for our pregnancies and little children.”*

(A mother-in-law from FGD 2)

The mothers-in-law also believe that asking men to be more involved in MNCH is disrespectful and that increased male engagement may lead to divorce. Some mothers-in-law also think that men are less capable of taking care of children and household chores.

*“Young women nowadays have no respect for their husbands. I normally see my son sweeping while the wife is seated or browsing on her phone, and when I ask him, he tells me that the house was dirty that’s why he swept. I ask him why he married because that is his wife’s duty. That is where marriage challenges begin from, and that’s why they never last.”*

(A mother-in-law from FGD 2)

One discussant agreed:

*“According to our African culture and tradition, this is regarded as a woman’s job, not a man’s job. It is the wife’s responsibility to know if her husband’s clothes are washed, what he would wear, cleaning children’s clothes, cleaning the house and cooking…. It does not matter if you are pregnant or not. Nowadays, the wife does not respect her husband. I see my sons even taking their pregnant wives to the stream to fetch water, they clean utensils together, until me as his mother feels bad, I think maybe the wife has power over him or what? I feel bad for them and sometimes I call my sons to make them stop doing their wives’ duties.”*

(A mother-in-law from FGD 2)

#### 3.2.2. Mothers

Most mothers strongly emphasized the importance of greater involvement of their husbands or partners in MNCH.

*“I think men should be involved in pregnancy and household tasks. For me, I will feel very happy because it means he would help me with house chores whenever they are too many. At least he will understand when he finds some work not done. It would show that he loves and cares for me. He does not want me to get tired more than him.”*

(A mother from FGD 3)

*“I hear my mother-in-law and my own mother saying pregnancy and house chores are for women. Personally, I do not agree that these tasks should be for women only. I tell them that since we are married, the pregnancy is for both of us, and I think that men should play the same role as women because they make the women pregnant in the first place. Therefore, men must take part in caring for the pregnancy and their children. They also should provide money. Nowadays even women can work full-time in paid jobs, just as their husbands do. So why should women take on all these responsibilities.”*

(A mother from FGD 3)

Mothers felt that their husbands’ or partners’ engagement in MNCH brought (or would bring) them happiness and satisfaction and showed them (women) that they were loved.

*“My husband’s involvement would make me very happy. It will show that he wanted the pregnancy, and we are in this together.”*

(A mother from FGD 1)

One discussant agreed:

*“I was very happy to be accompanied by my husband during the clinic visits. I think it will lead to a happy family. The wife even feels strengthened when she sees her husband helping.”*

(A mother from FGD 5)

One discussant provided a slightly different account indicating that male engagement may bring happiness, but preferred the traditional male role (i.e., providing for the family).

*“If my husband is involved, I will be happy because it will show that he loves me, but it should not be his most important role. He should focus more on providing for the family. He should just be involved in a bit of the house chores so that he is not underrated in the community.”*

(A mother from FGD 2)

Some women also indicated male engagement is a way to reduce violence and promote more humane treatment of women and set example for future generations.

*“It [male involvement] will boost love and cooperation in the family. It will also reduce domestic violence to a certain degree. If the man finds that something is not done, he will understand that his wife was busy with other chores. So, if he helps, he will understand the extent of house chores, hence reducing violence at home.”*

(A mother from FGD 3)

*“I think involving my husband will also help the children to be responsible while setting a good example for generations to come.”*

(A mother from FGD 5)

### 3.3. Women’s Endorsement of Traditional Gender Roles

In addition to their experience, and view of male engagement in MNCH, all women in the FGDs were asked about their view on traditional masculinity, which may influence their view on how men should behave with respect to MNCH. When asked about their view of a ‘real man,’ three themes frequently emerged in all the FGDs: sexual power and fertility, the ability to provide for the family, and love and care for the family.

Demonstrating one’s sexual prowess and fertility was discussed as being associated with what it means to be a real man.

*“A real man has many responsibilities, but the main thing is his duties in the bedroom. He has to be perfect in the bedroom.”*

(A mother from FGD 2)

*“A man who does not satisfy his wife sexually is not a real man. Without satisfaction, then the wife will have reasons to go out to another man, a real man.”*

(A mother from FGD 3)

Participants also alluded to the societal view and the treatment that men receive who are not considered real men.

*“In my view, a real man is the one who is able to marry a wife and have a child and that is when he will be perceived as a real man. Having a wife alone does not make him a real man. Without the ability to create his image (a child) people will start saying he has a certain problem, and he is not a real man.”*

(A mother-in-law from FGD 2)

In addition to being fertile and having the ability to satisfy his partner’s sexual desire, being a real man is also associated with the ability to provide for the family. Statements such as, “*mwanaume ni kazi*” (meaning, hard worker who is a sole breadwinner of the family), “*mwanaume ni kichwa*” (meaning, provider and leader of the family), and “*mwanaume ni sauti, pesa ndio*” (meaning, financially stable), were commonly discussed.

*“A real man is the one who provides food, education for the children, and clothing in general. He should do all it takes to achieve that because those are the roles of a man in the house.”*

(A mother from FGD 3)

*“What I call a real man is the one who cares for his family, provides for them, clothes the children and the wife, and feeds the family so that there is love in the family.”*

(A mother from FGD 1)

Most women in the study also indicated that a real man is the one who cares and shows love to his children and wife and who is a role model for his children.

*“I support the other respondents. A real man is one who is responsible, cares for the family and ensures their love between his children and wife.”*

(A mother from FGD 4)

Another discussant agreed:

*“I will second what my sister just said. A real man cares for them [his family] and loves them and ensures there is peace.”*

(A mother from FGD 3)

Some women also indicated that a real man is the one who helps his wife during pregnancy.

*“To me, a real man is closer to his wife when she is pregnant…. A real man should help his wife because when the wife is either sick or absent, then he should do the household chores.”*

(A mother from FGD 2)

*“A real man should help his wife. For instance, if the doctor orders his wife to be on bed rest, then he should take up the responsibility to help his wife.”*

(A mother from FGD 1)

In general, there was a limited endorsement of hegemonic masculine beliefs among mothers compared to mothers-in-law. Hegemonic masculine beliefs were predominantly discussed by mothers-in-law. In the mothers-in-law FGDs, gendered divisions, including cultural constructs of masculinity and generational dynamics, may have important implications for the role men could potentially play in MNCH care.

*“A real man, when he arrives home (a cock crows from the background), he should be like that cock that has just crowed; talk in a commanding voice ordering things to be done in an appropriate way. Everyone in the family should know that he has arrived.”*

(A mother-in-law from FGD 1)

*“The husband should be corrective and rebuke his wife against bad-mouthing his community. That is when the real man in him manifests. He should also tell the wife about his family situation and tell her to accept the way it is because he is the one who has married her. That is now being a real man.”*

(A mother-in-law from FGD 2)

### 3.4. Women’s Recommendations on Male Engagement in MNCH

All women in the FGDs were also asked about their opinions regarding strategies to engage men in MNCH. Participants recommended the use of group-based and community-based programming approaches, including public meetings (i.e., barazas), working with religious and community leaders, using mass media, and economic opportunities (e.g., animal husbandry), as strategies to encourage male engagement in MNCH.

*“Projects such as chicken farming and dairy farming will make husbands more disciplined and responsible. These projects will also ensure the mothers and newborn babies will be well-fed with the outcomes of such projects.”*

(A mother-in-law from FGD 2)

*“Community health workers should hold chief barazas and invite health professionals to educate men in healthcare. If men follow the guidelines taught, their children will in turn learn the importance of healthcare from their parents.”*

(A mother from FGD 1)

*“Sometimes getting the men is usually very hard, so these health workers should start walking around. They should go to churches, funerals, etc. Because convincing the men to go to clinics is very difficult.”*

(A mother from FGD 2)

*“Church leaders should hold meetings in churches so as to educate men on child and mother healthcare. Village elders should also hold barazas to educate men.”*

(A mother from FGD 2)

## 4. Discussion

This is the first known study to examine women’s perspectives on increased male engagement in MNCH in western Kenya. Overall, the participants noted that most males hesitate to engage in MNCH. However, the findings showed that most women in the study, except mothers-in-law, favored male engagement in MNCH and expressed the need to ensure gender-equitable engagement in order for women and children to benefit from male engagement. This study also showed the stark difference between mothers-in-law and mothers regarding male engagement in MNCH. Specifically, mothers-in-law generally endorsed a traditional view of masculinity and gender role differentiation (and favored less male involvement), whereas mothers preferred meaningful or gender-equitable involvement. Below, each of these major findings is described in detail.

Most of the participants, except for mothers-in-law, not only emphasized the importance of male engagement but also the need to ensure an equitable share of MNCH-related tasks by involving women in the decision-making process, including decisions during pregnancy. In other words, women desire male engagement approaches that support MNCH and gender equity. This is in accord with prior studies, which suggest the need to challenge gender inequity through male engagement in MNCH [22,23]. According to prior studies, male engagement approaches that focus mainly on men’s behaviors (and ignore their internalized identities, attitudes, and subjective experiences) are unlikely to create opportunities to engage with men to reform gender relations [23]. Such an approach neither views men as potential agents of positive change nor does it support transformative social change to challenge gender inequity.

Women who favored increased male engagement in MNCH widely discussed sociocultural factors as reasons for men’s reluctance to engage in MNCH. Several participants reported negative social perceptions towards men’s engagement in MNCH. For instance, most women in the FGDs reported that men who escorted their partners to MNCH services were perceived as being dominated, bewitched, or controlled by their wives. Women reported that this cultural belief not only led men to view MNCH care as designed and reserved for women but also led women to avoid engaging their male partners in MNCH. This finding supports the previous report on male engagement in MNCH care in Accra, Ghana, which found that men were concerned about how relatives and other people perceived them if they were seen getting involved in MNCH [24]. Furthermore, a study in Kenya found that male partners were seen as being controlled by their partners if they became involved in MNCH, which is also in line with the negative social perceptions towards men attending MNCH care services [25]. Such prohibitive traditional norms and gender roles are barriers to gender-equitable male engagement in MNCH [26]. The perception that a husband who engages in MNCH would be seen as being ruled by his wife may well be a sign of societal resistance against women’s empowerment or women being in charge, which, in turn, may perpetuate women’s subordination in society leading to negative health outcomes for women and their children.

In addition, some women in the study indicated that they would not want their husbands to be engaged in MNCH due to similar negative social perceptions. For example, women did not want to be stereotyped as ‘controlling’ wives who use sacrilegious means to control their husbands. Furthermore, the findings clearly show that women recognize both the potential benefits of increased male engagement in MNCH and the social costs that come with increased male engagement in MNCH. These social costs may include losing the opportunity to engage in social conversations and social learning with other women, and men dominating women’s traditional sphere of control. Such costs can be high enough to make women less receptive to increased male engagement in MNCH. These findings are consistent with prior quantitative studies conducted among males in Ghana that reported gender role conflicts, the discomfort that male engagement may bring, and negative stereotyping as some of the reasons why women are resistant to greater male engagement in MNCH [3,26].

The view of mothers-in-law in the study supported gendered divisions of labor that place men and women in particular physical spaces: men at work to provide for the family, and women at home taking care of children and household duties. According to mothers-in-law and a few mothers in the study, the household is perceived as the woman’s main domain. Some of the reasons why mothers-in-law and a few mothers are less supportive of increased male engagement in MNCH relate to: (1) their view that women are ‘naturally’ closer to children, both physically and as a result of their gender, (2) perceptions that MNCH matters are women’s business, and (3) their view of men’s limited ability to assume a caretaking role. According to these women, men are designed, by nature or God, to be household heads and breadwinners, whereas women are designed to be caretakers. As such, men should not engage in women’s business and should focus on playing their role as household breadwinners.

It is clear from this current study that mothers-in-law can be complicit in maintaining strict gender divisions and generational power dynamics that prevent meaningful gender-equitable male engagement in MNCH. Studies from the region reported similar findings regarding the influence of senior women [27,28]. According to Aubel [27] and Dumbaugh et al. [28], in many patriarchal societies, senior women (e.g., grandmothers, mothers-in-law, aunts) have a significant amount of influence over MNCH practices.

One of the key findings of the current study is the intrinsic acceptance by study participants, especially mothers-in-law, of social constructions and representations of masculinity and the need to maintain and assert these social norms. They believe engaging men in what is traditionally a woman’s role is disrespectful to men and their manhood. They also believe that men are supposed to be the controller, the decision-maker, and the head of the household, and should act as such. This finding supports the decades-long debate by masculinity and gender theorists that women, especially those in patriarchal societies, often endorse and aid the appraisal and maintenance of hegemonic masculine norms and associated behaviors [13,27,28,29,30]. According to these theorists, women support and endorse the appraisal of the traditional construction of masculinity even though they are often subordinated under these norms. This may be attributed to what is characterized in prior studies as ‘bargaining with patriarchy’ [31]. This ‘patriarchal bargain’ may be bolstering women’s acceptance of the traditional gender norms that delineate roles and responsibilities based on gender and women’s resistance to men’s engagement in MNCH.

It is worth mentioning, however, that this gender-based role differentiation and women’s support for hegemonic masculinity is not shared among all women in this study, as illustrated by some of the responses from mothers in this study. Such statements may be giving us a glimpse of a cultural shift that is starting to take place as more women participate in the workforce outside their homes. Nevertheless, the findings of this study indicate the need to include mothers-in-law in efforts to influence male engagement in gender-transformative MNCH initiatives. It is important for senior women to understand the benefits of male engagement in MNCH and support their sons or grandsons in transforming men’s roles as fathers. It is equally important to recognize women’s fear of increased male engagement in MNCH as it potentially limits their freedom. Increasing men’s abilities to influence the activities and decisions that are traditionally dominated by women, especially in patriarchal societies like those in western Kenya, could lead to negative consequences for women. For example, it may increase men’s controlling behavior, decrease women’s decision-making power, create discomfort for women, or increase men’s use of force against their partner. Thus, any effort to increase male engagement in MNCH should ensure that it does not lead to undesirable shifts in power and inadvertently result in the disempowerment of women [32]. Prior studies reported women’s concern regarding male involvement in creating inequalities and preferential treatment in the service delivery process [3,33].

It is also important to note that mothers in this study who were supportive of increased male engagement provided recommendations to change the social norms that are barriers to increasing male engagement in MNCH. The recommendations included four major approaches: (1) engaging men early on using group-based and community-based approaches; (2) engaging influential figures in the community, such as community and religious leaders to educate and motivate men to engage in MNCH; (3) using economic development strategies to target men; and (4) using media, such as radio and television, to educate men and mothers-in-law on the benefits of their engagement in MNCH. Prior studies reported the importance of such a multipronged approach to increasing male engagement in MNCH [34,35]. It is worth noting, particularly, that the recommendations included engaging men early in the life course or when they are young boys. Starting with young boys and helping them to engage in MNCH can have profound impacts, as these boys will likely have a greater chance of having more equitable relationships and positively influencing those around them for the rest of their lives [36].

### 4.1. Limitations

As a qualitative study, the ability to produce rich descriptions and explore nuances regarding women’s views of increased male involvement in MNCH and related social norms is a strength of this study, but also a limitation, in its use of ethnically and socioeconomically homogeneous groups from a relatively small study area. A second limitation of this study is the potential for effects of age hierarchy in conversation and dominant discussants, which may have biased to content of the FGDs. Despite the limitations, the findings demonstrate how and to what extent women want men to be involved in MNCH. This study also expands on the existing literature by expanding the understanding of masculine values within MNCH research.

### 4.2. Programmatic Implications

Taken together, the study sheds light on the following key findings and implications for program design and intervention:

1. Diverse Perspectives Among Women: This study highlights that there is no uniform perspective among women regarding increased male engagement in MNCH. Women’s views and attitudes are shaped by various factors, including their roles, generational differences, gender norms, and cultural beliefs.
2. Women’s Support for Gender-Equitable Male Engagement: Except for mothers-in-law, women in this study, were supportive of gender-equitable male involvement in MNCH. They recognized the importance of joint decision making and a more balanced sharing of responsibilities in child-rearing. This suggests that some women are open to redefining traditional gender roles, especially the younger generation.
3. Mothers-in-law’s Resistance and Influence: Mothers-in-law, who often hold influential roles within families, were less supportive of increased male involvement. They expressed concerns that MNCH is primarily a woman’s responsibility, and they were hesitant about men taking on a more active role in MNCH. Some believed that men’s involvement could have negative consequences for children and women. The study underscores the significant influence that mothers-in-law can have on family dynamics and decisions, particularly regarding their sons’ marriages and fatherhood. Their resistance to male involvement in MNCH could impact the success of programs promoting such involvement.
4. Programmatic Considerations: The findings suggest that programmatic initiatives aimed at enhancing male engagement in MNCH must consider the perspectives and concerns of women. Ignoring these concerns could hinder the effectiveness of such initiatives. Involving mothers-in-law in the design and implementation of programs not only helps address their apprehensions but also ensures their important influence and role in MNCH as trusted advisors and alternative caregivers are acknowledged. For example, a study from Kenya highlighted the importance of engaging influential family members to support child nutrition by leveraging grandmothers’ roles as advisors and promoting gender-transformative activities [37].
5. Opportunities for Gender-Equitable Male Engagement: This study also identifies an opportunity to make a change by leveraging the influence of mothers who are supportive of gender-equitable male engagement. These women can serve as advocates for change and play a role in promoting more balanced and shared responsibilities in MNCH.

## 5. Conclusions

This study highlights the importance of considering the diverse perspectives of women, both mothers and mothers-in-law, when developing strategies to engage men in MNCH. It emphasizes the need for gender-equitable male involvement that fosters joint decision making and shared responsibilities while ensuring a safe cultural environment (e.g., receiving buy-in from older generations). Additionally, it underscores the need to address and work with the concerns of mothers-in-law, who can exert significant influence over family dynamics, to ensure the success of MNCH initiatives.

## Figures and Tables

**Table 1:** The extent of the desirability of male engagement by women.

Type of Male Involvement	Mothers-in-Law	Mothers
Accompanying wives to MNCH care (ANC, SBA, PNC)	Desirable for few	Very desirable for many
Providing financial support	Very desirable for all	Desirable for many
Taking care of children (bathing, changing diapers etc.)	Not desirable	Desirable for many
Assisting in household chores (cooking, cleaning, etc.)	Not desirable	Desirable for many
Joint decision-making	Not desirable	Very desirable for all
Overall increased involvement	Not desirable	Very desirable for many

## Data Availability

The data presented in this study are available on request from the corresponding author due to the sensitive nature of the focus group discussion transcripts.

## Supplementary Materials

The following supporting information can be downloaded at: https://www.mdpi.com/article/10.3390/ijerph22010125/s1, Annex S1: English Version of the Focus Group Discussion Guide.
