# Supplementary material for: “My Husband Is a ‘Mama’s Boy’”: Women’s Views on Male Engagement in Maternal, Newborn, and Child Health in Western Kenya
Source: Int J Environ Res Public Health. 2025 Jan 19;22(1):125. doi: 10.3390/ijerph22010125 (PMC11765237; doi:10.3390/ijerph22010125)
Supplement: Supplementary file 1 [file ijerph-22-00125-s001.zip › ijerph-3269751-supplementary.pdf]

QUALITATIVE INTERVIEW GUIDE FOR FOCUS GROUP DISCUSSIONS

Thank you again for agreeing to participate in this interview. I would like to remind you that this interview is confidential and private and will not have any impact on your ability to access health services in the future.

1. Experience with and view about male involvement in MNCH

Now I would like to ask you a few questions about your experience of your husband's/partner's involvement in your health care as a mother and in your child's health care.

1.1 Tell me about your experience going to health care visit for childbirth and after the birth of your child

> *1.1.1 Did your husband/partner ever go to health care visits with you?*
> *[if yes] how did you feel about it?*
> *[if no] why do you think he did not? How did you feel about it?*
>
> *1.1.2 What do you think about men becoming more involved in activities such as taking care of your child (e.g., changing diapers, giving the child a bath, babysitting, taking the child to health facility), cooking for you and the children, fetching water, cleaning house, doing laundries?*

2.2 [Note to the interviewer: Ask this question if the respondent said "Yes" to question 2.1.1] Tell me how other people felt about your husband accompanying you to healthcare visit.

> *2.2.1 What did your friends say?*
>
> *2.2.2 What did your family say?*
>
> *2.2.3 What did your mother-in-law say?*
>
> *2.2.3 What did community members say?*

2.2 [Note to the interviewer: Ask this question if the respondent said "No" to question 2.1.1] Tell me how other people felt about your husband not accompanying you to healthcare visit.

> *2.2.1 What did your friends say?*
>
> *2.2.2 What did your family say?*
>
> *2.2.3 What did your mother-in-law say?*
>
> *2.2.3 What did community members say?*

2.3 Tell me how other people would feel if your husband accompanied you to health visits

> *2.3.1 What would your friends say?*
>
> *2.3.2 What would your family say?*
>
> *2.3.3 What would your mother-in-law say?*
>
> *2.3.3 What would community members say?*

2.4 How much do you worry about what other people might say about your husband doing these activities?

2. Endorsement of traditional masculinity and gender roles

You are doing great. Thank you for answering these questions. Now I would like to ask you a few questions about your opinion regarding how men are viewed.

3.1 What does a man have to do for others (including his wife) to consider him a real man?

3.2 What do you think about these characteristics?
Probe: Do you agree with them? Tell me more why or why not?

You are doing great. Thank you for answering these questions. Now I would like to read a scenario to you and I would love to hear your response to these scenarios

3.3 Some people say "real men don't get involved in women's work such as taking care of children, doing laundries, cooking, taking his wife to health facility etc." Do you support such views?

[if yes] tell me tell me why you think real men do not do these activities
[if no] tell me tell me why you do not support the views

3.4 *How would you respond to someone who says "Men's greater political, economic, decision-making, and social power over women is natural or God-given and it should not and will not change?"*

Probe: Tell me more
3.5 How would you respond to someone who says, "household decisions should be made only by the man and not the woman?"
Probe: Tell me more

***3.* Women's recommendation regarding male involvement intervention**

Now, I would like to ask one final question about your opinion regarding male involvement in your health care as a mother and in your child's health care

3.1 How do you think health workers (nurses, doctors, public health professionals, and community health workers) can encourage men to be involved in wives and child healthcare and involve women in decision-making at household level?
3.2 How do you think elected officials can encourage men to be involved in their wives and child healthcare and involve women in decision-making at household level?
3.3 How do you think charity or NGO workers can encourage men to be involved in their wives and child healthcare and involve women in decision-making at household level?

4. Anything you want to add?

Thank you for your time and participation!

Note for interviewer: Try to use the following probes as appropriate

- Uh-huh (affirmative listening)
- Tell me more
- What does that mean to you?
- If I understand correctly… (Repeating back a summary of the participant's statement)
- It sounds like you were saying you……… (Rephrase the statement)
- Nods head but remains silent
- How so?
- Why is that?
- Could you give me an example?
